# Guanidine-modified albumin-MMAE conjugates with enhanced endocytosis ability

**DOI:** 10.1080/10717544.2023.2219433

**Published:** 2023-07-11

**Authors:** Ce Yi, Fei Xie, Xin Xu, Dian Xiao, Xinbo Zhou, Maosheng Cheng

**Affiliations:** a School of Pharmaceutical Engineering, Shenyang Pharmaceutical University, Shenyang, China; b National Engineering Research Center for the Emergency Drug, Beijing Institute of Pharmacology and Toxicology, China Beijing

**Keywords:** Albumin drug conjugates, guanidine modified, endocytosis ability, improve efficacy

## Abstract

Aiming to address the insufficient endocytosis ability of traditional albumin drug conjugates, this paper reports elegant guanidine modification to improve efficacy for the first time. A series of modified albumin drug conjugates were designed and synthesized with different structures, including guanidine (GA), biguanides (BGA) and phenyl (BA), and different quantities of modifications. Then, the endocytosis ability and *in vitro*/*vivo* potency of albumin drug conjugates were systematically studied. Finally, a preferred conjugate A4 was screened, which contained 15 BGA modifications. Conjugate A4 maintains spatial stability similar to that of the unmodified conjugate AVM and could significantly enhance endocytosis ability (p*** = 0.0009) compared with the unmodified conjugate AVM. Additionally, the *in vitro* potency of conjugate A4 (EC_50_ = 71.78 nmol in SKOV3 cells) was greatly enhanced (approximately 4 times) compared with that of the unmodified conjugate AVM (EC_50_ = 286.00 nmol in SKOV3 cells). The *in vivo* efficacy of conjugate A4 completely eliminated 50% of tumors at 33 mg/kg, which was significantly better than the efficacy of conjugate AVM at the same dose (P** = 0.0026). In addition, theranostic albumin drug conjugate A8 was designed to intuitively realize drug release and maintain antitumor activity similar to conjugate A4. In summary, the guanidine modification strategy could provide new ideas for the development of new generational albumin drug conjugates.

## 1. Introduction

Small molecule chemotherapy drugs play an important role in tumor treatment, but their short half-life and systemic toxicity limit their clinical use (Yousefpour et al., 2019). As an excellent drug delivery carrier, human serum albumin (Alb) has been widely applied in the development of anticancer drugs (Tao et al., 2021). The advantages of Alb include good tolerance, nontoxicity, low immunogenicity, a long circulating half-life (approximately 19 days) (Alvarez et al., 2010; Sleep et al., 2013), and wide applicability (Kratz, 2008).

At present, drugs with albumin as a carrier mainly include albumin drug conjugates, albumin physical embedding drugs and albumin-modified carriers (Fiehn et al., 2008; Li et al., 2008; Zu et al., 2009; McCann et al., 2010). Among them, research on albumin drug conjugates has been the most in depth. The first strategy for research on albumen drug conjugates is albumin-bound drugs, which need to be conjugated with albumin *in vivo*. For example, Aldoxorubixin (AlDox, marketed in 2014) (Kratz, 2007; Gong et al., 2018) covalently combines its maleimide group with cysteine (Cys34) of albumin *in vivo* (Fiehn et al., 2008). In the acidic tumor microenvironment, the hydrazone bond of AlDox’s linker is cleaved to release doxorubixin (Dox) to exert an antitumour effect. However, the development of this drug was suspended because of cardiac toxicity, which could be caused by AlDox’s failure to fully bind albumin *in vivo*. The second strategy is albumin fusion peptide drugs. For example, exenatide and human serum albumin fusion protein (E2HSA) are designed to treat diabetes, and phase II clinical trials have been completed. The third strategy is albumin drug conjugates (*in vitro*). Fiehn C et al. (Fiehn et al., 2008) covalently conjugated methotrexate (MTX) with albumin *in vitro* to obtain MTX-HSA through a linker, which can achieve the same effect in the collagen-induced arthritis mouse model when the dosage is only 20% of free MTX. Until now, the latest research progress of albumin drug conjugates was that of Xinquan Liu et al. (2020) They tried to replace the antibody of ADC (antibody drug conjugate): Adcetris (CD30-VC-MMAE, VC-MMAE is the most successful linker payload in ADC) with albumin. The obtained conjugate Alb-VC-MMAE was broad-spectrum compared with Adcetris and could avoid the need for high antigen expression. Alb-VC-MMAE retains the linker as a VC dipeptide structure sensitive to cathepsin B (CTSB), and the payload is MMAE. However, to achieve similar *in vivo* potency, Adcetris (MMAE: 0.018 mg/kg) was significantly greater than conjugate Alb-VC-MMAE (Conjugate: 47 mg/kg, MMAE:0.5 mg/kg). In summary, although albumin as a broad-spectrum drug carrier could obviously reduce the times of administration, its weak efficacy still limits its use. It is speculated that one of the main reasons for albumin’s weak efficacy is insufficient endocytosis, which reduces bioavailability (Postupalenko et al., 2015; Ixon et al., 2016).

**Figure 1.**
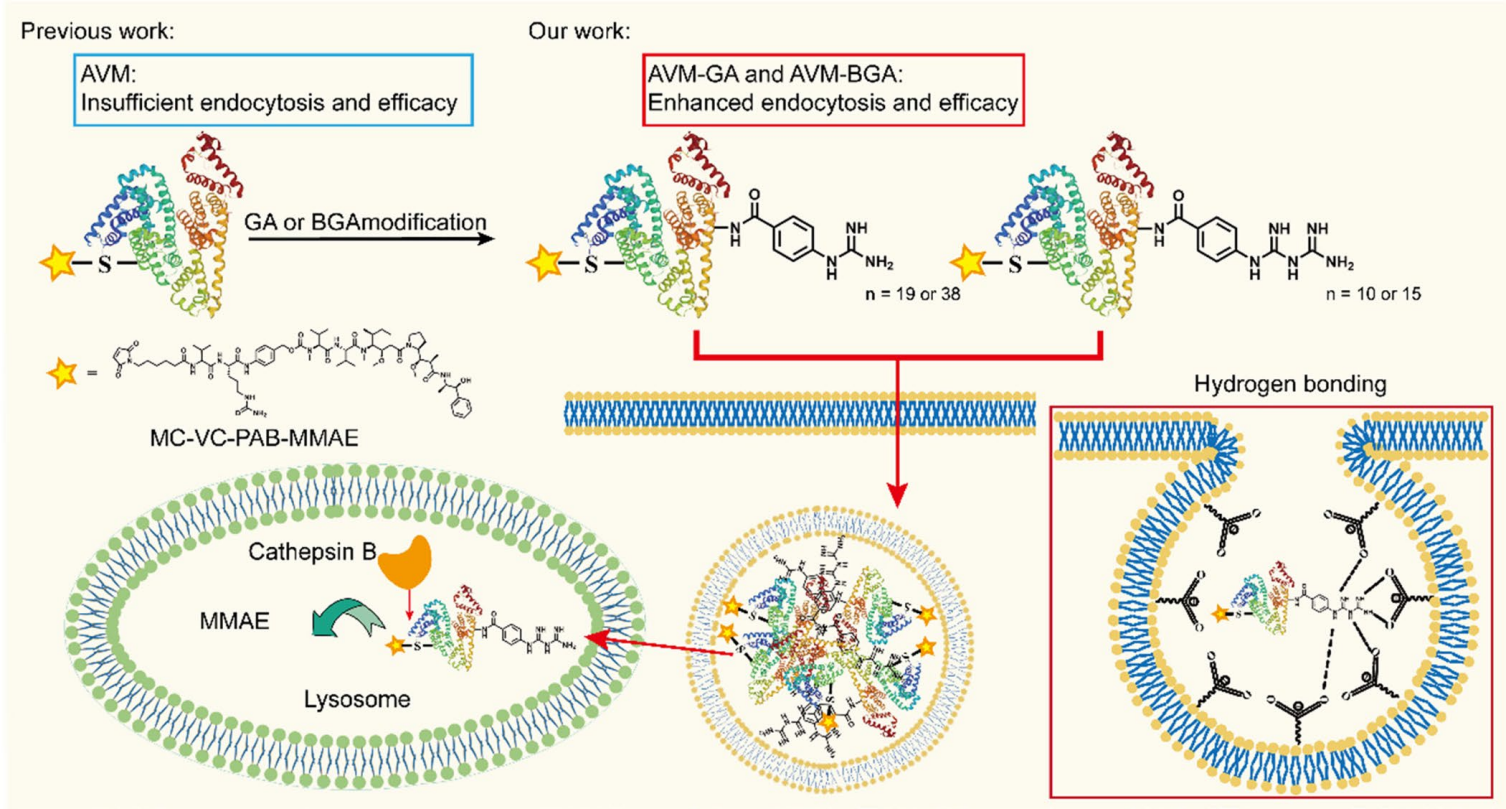
the structure of chemical small molecule and introduce of previous work and our work.

Therefore, a new strategy has been reported to enhance the endocytosis ability of guanidine-modified albumin drug conjugates for the first time. Guanidine modification technology is an extensive strategy to increase the endocytosis of macromolecules (Kurzawa et al., 2010). The phosphate group on the cell membrane is a negative environment, meanwhile the guanidine-modified albumin could enhance the positive electricity. Therefore, both of them will be attracted each other under electrostatic action and then formed hydrogen bond to strengthen endocytosis (Ikeda et al., 1984; Martin et al., 2008). The advantage of guanidine modifications, such as guanidine (GA) and biguanide (BGA) (Choi et al., 2004; Posey et al., 2018), might be controllable quantities of connections and a larger area distribution, which could increase flexibility to form hydrogen bonds with the cell membrane. It is satisfactory that albumin has enough modification sites (60 lysine), and in order to increase the quantities of guanidine modification, the albumin part of the conjugate is selected to modify. In brief, guanidine-modified conjugates were designed and synthesized with different structures and quantities. Then, the endocytosis ability and *in vitro/vivo* activity were systematically compared between the modified and unmodified conjugates. Finally, the preferred conjugate A4 was screened, which markedly enhanced endocytosis and *in vitro/vivo* activity. Eventually, the guanidine modification strategy could provide new ideas for the development of new generational albumin drug conjugates (Figure 1).

## 2. Materials and methods

### 2.1. Materials

All experimental reagents and solvents were purchased from Innochem, Energy Chemical, Aladdin, Macklin, etc. and human serum albumin was bought from ZHEJIANG HISUN Pharmaceutical CO., LTD. All experimental reagents and solvents were directly used without further purification.

### 2.2. Synthesis of compounds

(1) Key intermediate compound 2–4

The compound 2–4 were synthesized as previously reported (Zhang et al., 2019).

(2) Conjugate AVM (5)

Human serum albumin solid powder is dissolved into PBS to prepare a 5 mg/mL solution (pH = 7.4). And then compound 4 (3 equivalents) was added to the solution in dimethylacetamide (DMAC) (8% v/v, ice-cold). Maintain reaction for 12 hours, the reaction mixture was eluted through TSKgel Phenyl-5PW for removing unreacted albumin and other substances. And for PBS exchange, the Sephadex-G25S was used to eluted the mixture. To further ensure the quality of the conjugates, 0.2 μm filter were used to sterile-filtered and purified conjugates stored at −80 °C for future use.

(3) Key intermediate compounds 10–12

The compounds 6–8 (0.15 mmol) were dissolved in 1 mL N,N-Dimethylformamide and then triethylamine (0.294 mmol) and compound 9 (0.285 mmol) was added in turn, and contain reaction for 1 h at room temperature, after that reduce the temperature to 0 °C and contain reaction for 1h. Then the reaction solution was centrifuged at 4000 rpm for 15 min and take the supernatant at 0 °C for storage.

(4) Conjugates A1–A6

The conjugate 5 (5.6 mg/mL, 1.2 mL in PBS) was exchange to 2 mL CBS (pH = 9.6) buffer by centrifuge at 9000 rpm for 10 min, then 19.5 μL (to synthesis conjugate A1, A3 and A5) or 39 μL (to synthesis conjugate A2, A4 and A6) solution of compounds 10–12 were added and maintain the reaction for 12 h at room temperature. And for PBS exchange, the Sephadex-G25S was used to eluted the mixture. To further ensure the quality of the conjugates, 0.2 μm filter were used to sterile-filtered and purified conjugates stored at −80 °C for future use.

(5) Key intermediate compound E-15C

The compound E-15C were synthesized as previously reported (Xiao et al., 2021) and the synthesis route has been stated in the supplementary materials.

(6) Conjugates A7 and A8

Human serum albumin solid powder is dissolved into PBS to prepare a 5mg/mL solution (pH = 7.4). And then compound E-15C (3 equivalents) was added to the solution in dimethylacetamide (DMAC) (8% v/v, ice-cold). Maintain reaction for 12 hours, the reaction mixture was eluted through TSKgel Phenyl-5PW for removing unreacted albumin and other substances. And for PBS exchange, the Sephadex-G25S was used to eluted the mixture. To further ensure the quality of the conjugates, 0.2 μm filter were used to sterile-filtered and purified conjugates stored at −80 °C for future use.

The conjugate Alb-E-15C (5.6 mg/mL, 1.2 mL in PBS) was exchange to 2 mL CBS (pH = 9.6) buffer by centrifuge at 9000 rpm for 10 min, then 39 μL solution of compound 18 was added and maintain the reaction for 12 h at room temperature. Then the mixture was placed on ice for 30 min and then eluted through Sephadex-G25S for PBS exchange. To further ensure the quality of the conjugates, 0.2 μm filter were used to sterile-filtered and purified conjugates stored at −80 °C for future use.

### 2.3. Quality control

To determine the molecular weight of the conjugates, 5800 TOF/TOF (AB Sciex, Framingham, MA, 01701, USA) instrument was used. And the setting parameters of the instrument are as follows: Calibration batches was used as internal calibration to run instruments, which mass tolerance was within the range of ±800 ppm. The minimum signal to noise ratio was set to 4, and the maximum outlier error of the result is in the range of 500 ppm. The reference masses were used as follow: 33216 m/z (BSA++) and 66431 m/z (BSA+). 10000 to 100000 m/z as the mass range of acquisition methods, and 60000 m/z as the focus mass. The laser intensity was 7500, and there are a total of 4000 shots per spectrum. And to process spectra, baseline subtraction (peak width 30) was used. A minimum signal to noise ratio was set to 3, and peak detection with the local noise window width was set to 400 m/z. The resolution was set to 200. The above experimental methods refer to previously reported (Huber et al., 2019).

Circular dichroism (CD) spectra were recorded by Biologic MOS-450 (Claix, France) CD Spectrometer. Samples were diluted to 11 μM in PBS and measured in a rectangular quartz cell (pathlength 1 mm) sealed with a Teflon stopper. The CD spectra was recorded in the range from 260 nm to 180 nm with 1 nm step and 0.5 s sampling time.

The UNcle running the Client software V5.03 to test the Dynamic light scattering (DLS), which laser was 660 nm. Before filling the sample into the loader, ensure that the volume of each sample is 9 μL. During the measurement process, the data was collected for an average of four/5 seconds, and the laser power auto-attenuated after each acquisition was beginning. The distribution fit of the preset general-purpose analysis model was used to conduct size analysis (Kannan et al., 2022).

T_m_ and T_agg_ data were acquired by the Uncle, which running the Client software V5.03. A linear thermal ramp was performed from 15 to 95 °C at a rate of 0.3 °C/min. Before filling the sample into the loader, ensure that the volume of each sample is 9 μL. UV Filter 1 and Blue Filter 3 were used to illuminated the samples, which incubation time was 120 seconds. And then UNcle Analysis software V5.03 achieved Data analysis. Through analyzed the average center of gravity (BCM) between 300 and 430nm, the changes in intrinsic fluorescence with temperature were monitored, which excited using the 266 nm laser. Under appropriate conditions, detected changes in static light scattering by measuring the intensities of the peaks at 266 and 473 nm. At last, through identified by the differential of the maximum gradient of the BCM versus temperature traces to obtained T_m_ values. The above experimental methods refer to previously reported (Huang et al., 2022).

### 2.4. Cytotoxicity test

Cells in 100 μL complete medium were plated and incubated under growth conditions in 96-well plates which density was 5 × 10^3^ cells per well. After 24 h of plating, all samples were gradiently diluted 3-fold into multiple concentration gradients and added to each well. After 96 h of incubation in incubator, plates were taken to equilibrated at room temperature for 0.5 h. And then, 100 μL Cell Luminescent Solution was added to each well. The plates kept shaking for 5 mins at 650 rpm, and which were placed in room temperature for 10 mins. At last, Luminescence intensity was measured on a GloMax® Navigator system, which integration time of 1 second per well. The above experimental methods refer to previously reported (Xiao et al., 2021).

### 2.5. Cell apoptosis and cell cycle analysis

To evaluate cell apoptosis, SKOV3 cell lines in 100 μL complete medium were plated in 12-well plates at a density of 5 × 10^4^ cells/well. Conjugate A4 at various concentrations (0, 100 and 300 nM) were added in each well and incubated under growth conditions for 48 h. To detected apoptosis and cell death, Annexin V-FITC Apoptosis Kit (Dojindo, Japan) and propidium iodide (PI) were used to stain, and the fluorescence intensity were measured by BD FACSAria II.

To evaluate cell cycle, SKOV3 cell lines in 100 μL complete medium were plated in 12-well plates at a density of 5 × 10^4^ cells/well. Conjugate A4 at various concentrations (0, 100 and 300 nM) were added in each well and incubated under growth conditions for 48 h. And bromodeoxyuridine (BrdU; Beyotime Biotechnology, Shanghai, China) was added and incubated for 20 min. To detected Nascent DNA synthesis and total DNA content, anti-BrdUrd FITC and phycoerythrin (PE) were used, respectively. To detected cell cycle position, FACSCalibur (BD Biosciences) was used. The above experimental methods refer to previously reported (Xiao et al., 2021).

### 2.6. In vivo efficacy studies

Female BALB/c nude mice at 6–8-week-old were obtained from SPF (Beijing) biotechnology co., LTD. All mice were kept as 3 per cage in SPF Class Animal Feeding Center after unpacked. Moreover, ARRIVE guidelines 2.0 were followed for animal study. Before the experiment, the experimental scheme was determined that mice bearing SKOV3 cells xenografts mice were randomized when the tumor sizes were about 120 mm^3^, respectively. 6 BALB/c nude mice were used in each group for experiments, and their weigh between 16 and 23 g. The mental state, activity, hair color and body weight of all mice was observed and recorded in first week. AVM (47 mg/kg) were administered through tail vein every 7 days for a total of 4 doses in SKOV3 cells xenografts. PBS vehicle controls were also administered. Tumor sizes and body weight were measured by a digital caliper twice a week starting from Day 0. Mouse food and tap water was provided to the animals. SKOV3 cells were harvested during the logarithmic growth phase and resuspended in sterile saline before inoculation in animals. 1 × 10^7^ cells were inoculated subcutaneously into mice. And measured the tumor volume and body weights every two weeks, and the equation (0.5×L × W^2^, L = the length of tumor, W = the width of tumor) was used to calculated tumor volume. At the end of the experiment, the mice were euthanized by cervical dislocation after anesthesia, with a dose of 10 μL/g (Pentobarbital sodium).

Female BALB/c nude mice at 6–8-week-old were obtained from SPF (Beijing) biotechnology co., LTD. All mice were kept as 3 per cage in SPF Class Animal Feeding Center after unpacked. Moreover, ARRIVE guidelines 2.0 were followed for animal study. Before the experiment, the experimental scheme was determined that mice bearing SKOV3 cells xenografts mice were randomized when the tumor sizes were about 180 mm^3^, respectively. 6 BALB/c nude mice were used in each group for experiments, and their weigh between 16 and 23 g. The mental state, activity, hair color and body weight of all mice was observed and recorded in first week. AVM (17 mg/kg), AVM (34 mg/kg), A4 (17 mg/kg), and AVM (34 mg/kg), were administered through tail vein every 4 days for a total of 3 doses in NCI-N87 xenografts. PBS vehicle controls were also administered. Tumor sizes were measured by a digital caliper twice a week starting from Day 0. Mouse food and tap water was provided to the animals. NCI-N87 cells were harvested during the logarithmic growth phase and resuspended in sterile saline before inoculation in animals. 1 × 10^7^ cells were inoculated subcutaneously into mice. And measured the tumor volume and body weights every two weeks, and the equation (0.5×L × W^2^, L = the length of tumor, W = the width of tumor) was used to calculated tumor volume. At the end of the experiment, the mice were euthanized by cervical dislocation after anesthesia, with a dose of 10 μL/g (Pentobarbital sodium).

### 2.7. Stability of conjugate A4

The stability of conjugate A4 in human plasma and PBS were determined by LC-MS, the standard sample was prepared through MMAE diluted into human plasma in equal volume, and the standard sample of conjugate A4 was prepared at same as method. All samples were incubated in constant temperature incubator for 10 days at 37 °C. Through LC-MS, MMAE standard curve was established. And which contributed to detected the concentration of total free MMAE released from conjugate A4 at various time-points by LC-MS. The above experimental methods refer to previously reported (Xiao et al., 2021).

### 2.8. Fluorescence imaging

SKOV3 cells and HeLa cells were grown in culture medium, the specific cultivation conditions were as follows: the temperature was 37 °C, atmosphere at 5% CO_2_, and Dulbecco’s Modified Eagle and RPMI-1640 medium were used respectively, which needed 10% fetal bovine serum and 0.1% penicillin-streptomycin to added. SKOV3 cells or HeLa cells were both seeded in the chamber at a density of 3 × 10^4^ cells per well and incubated overnight. The Alb-FITC, Alb-BGA15-FITC, AVM-FITC and A4-FITC (600 nM) were added into cells and incubated for 24 h. After that, the PBS solution was used to wash cells briefly, and then Lyso-Tracker and Hoechst-Tracker were added into the media. Then PBS solution was used to wash cells three times, after that the cells were placed in 1 mL of PBS solution. At last, Fluorescence images were detected by a confocal laser scanning microscope (Nikon ECLIPSE Ti, Nikon, Japan). The above experimental methods refer to previously reported (Xiao et al., 2021).

SKOV3 cells was grown in culture medium, the specific cultivation conditions were as follows: the temperature was 37 °C, atmosphere at 5% CO_2_, and Dulbecco’s Modified Eagle and RPMI-1640 medium were used respectively, which needed 10% fetal bovine serum and 0.1% penicillin-streptomycin to added. SKOV3 cells or HeLa cells were both seeded in the chamber at a density of 1 × 10^5^ cells per well and incubated overnight. The AVM-FITC and A4-FITC (600 nM) were added into cells and incubated for 24 h. After that, the PBS solution was used to wash cells briefly, and Trypsin-EDTA used to prepare cell suspension and centrifuge, then remove the supernatant and wash it with PBS three times, the cells were placed in 200 μL of PBS solution. Flow cytometric analysis were *t* detected by BD FACSAria II.

### 2.9. Spectroscopic materials and methods

Stock solutions of biologically relevant analytes (NaCl, KCl, CaCl_2_, glucose, MgCl_2_, Glu, VC, Arg, Ser, GSH, Cys, DTT, H_2_O_2_, NaClO, and CTSB.) were prepared in PBS buffer. All spectroscopic measurements were performed in a simulated lysosomal environment (Cathepsin B activity buffer containing 10% (v/v) DMSO, pH 5.0 or 6.0, 37 °C). Both absorption and fluorescence spectra were recorded using a microplate reader. Samples for absorption and emission measurements were obtained from wells of 96-well plates. Excitation was carried out at 373 nm.

## 3. Results and discussion

### 3.1. The design of albumin drug conjugates

Aiming to address the problem of insufficient endocytosis of traditional albumin drug conjugates, the idea of guanidine modification to enhance endocytosis was proposed. It was expected that hydrogen bonds could be formed between the guanidine group and cell membrane, which could enhance endocytosis. However, this strategy has not been applied in the research of albumin drug conjugates. Tao (2017) covalently linked biguanide to BSA and then loaded antitumor drugs through nanoparticles. The obtained conjugate could effectively enhance endocytosis and increase antitumor efficacy.

In this design, the guanidine modification, linker and payload of albumin drug conjugates have been systematically considered. For the albumin, different structures and amounts of guanidine modifications, such as GA and BGA, were introduced. For the payload, MMAE was selected, which is the commonly used payload in ADCs. For the linker, the VC dipeptide structure was chosen, which is sensitive to cathepsin, and notably, could release payload in the lysosomes of tumor cells.

### 3.2. Chemistry

**Scheme 1.**
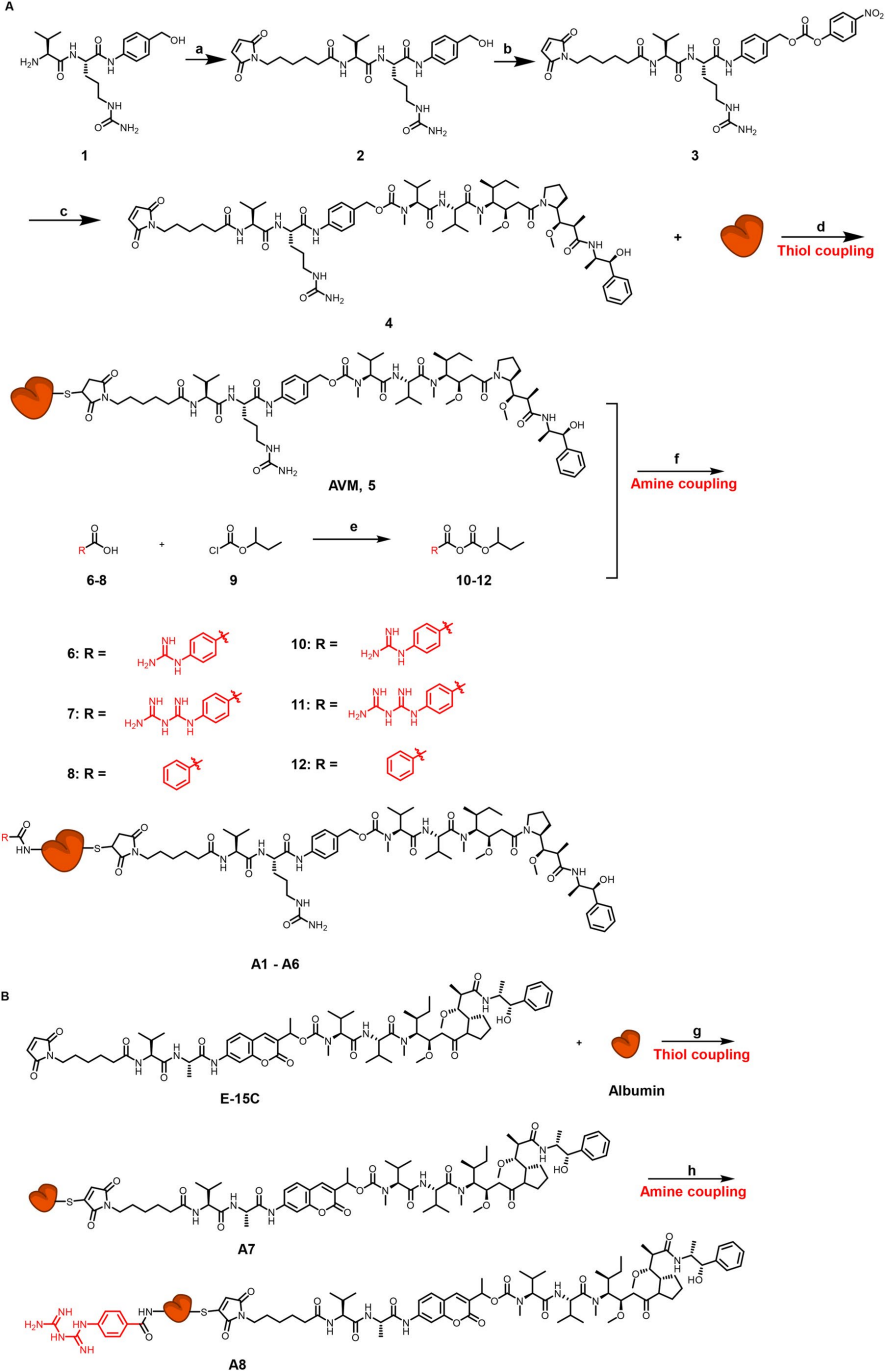
A) Synthesis of conjugates A1 – A6. Reagents and conditions: (a) N-Succinimidyl 6-maleimidohexanoate, DMF, r.t., overnight; (b) Bis (4-Nitrophenyl) Carbonate, DIPEA, DMF, r.t., 5 h; (c) MMAE, DIPEA, HOBt, DMF, r.t. overnight; (d) PBS, r.t., 12h; (e) TEA, r.t., 1h, 0°, 1h; (f) PBS, r.t., 12h; B) Synthesis of conjugates A7-A8. Reagents and conditions: (g) PBS, r.t., 12h; (h) compound 11, TEA, r.t., 1h, 0°, 1h.

The synthetic routes of conjugates A1–A8 are shown in Scheme 1. The key intermediate conjugate 5 (AVM) was obtained in the following steps. Compound 1 was converted into compound 2 through condensation reaction with N-succinimidyl 6-maleimidohexanoate. And compound 3 was obtained by reaction of compound 2 and bis (4-nitrophenyl) carbonate, then it reacts with MMAE and albumin to obtain the key conjugate AVM. To further synthesize conjugates A1 to A6, compounds 6–8 were reacted with isobutyl chlorocarbonate to give compounds 10–12, and finally conjugate 5 (AVM) was reacted with compounds 10–12 in different quantities to give the modified conjugates A1–A6 (Scheme 1A). The structure and quantities of small molecules (GA, BGA and BA) conjugated to AVM are shown in Table 1.

**Table 1.** Synthesis route and structure of conjugates A1–A6.

NO.	Conjugates name	Quantities of modifier	Structure of modifier (R)
A0	AVM	–	–
A1	AVM-GA	19	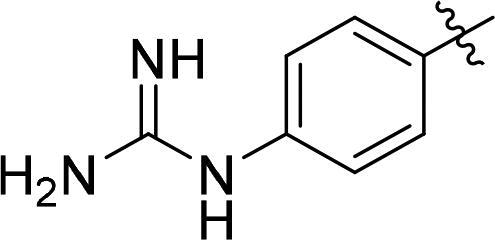
A2	AVM-GA	38	
A3	AVM-BGA	10	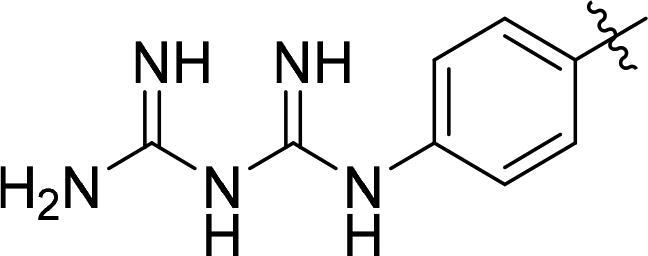
A4	AVM-BGA	15	
A5	AVM-BA	13	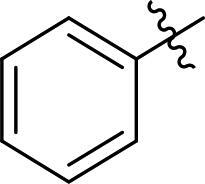
A6	AVM-BA	28	
A7	Alb-E-15C	0	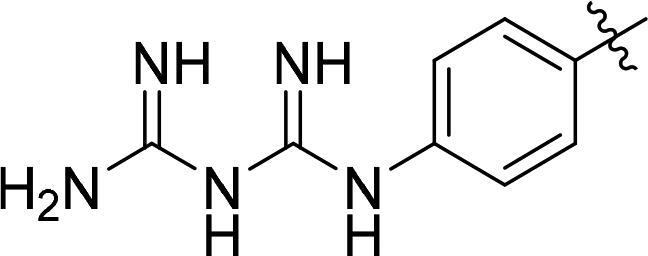
A8	Alb-E-15C-BGA	15	

The synthesis route of compound E-15C has been stated in the supplementary materials (Figure S2). Conjugate A7 was obtained by compound E-15C and Alb conjugated in PBS. Then, compound 11 was reacted with conjugate A7 to give modified conjugate A8 (Scheme 1B).

### 3.3 Study on the structure-activity relationship of guanidine-modified AVM

**Figure 2.**
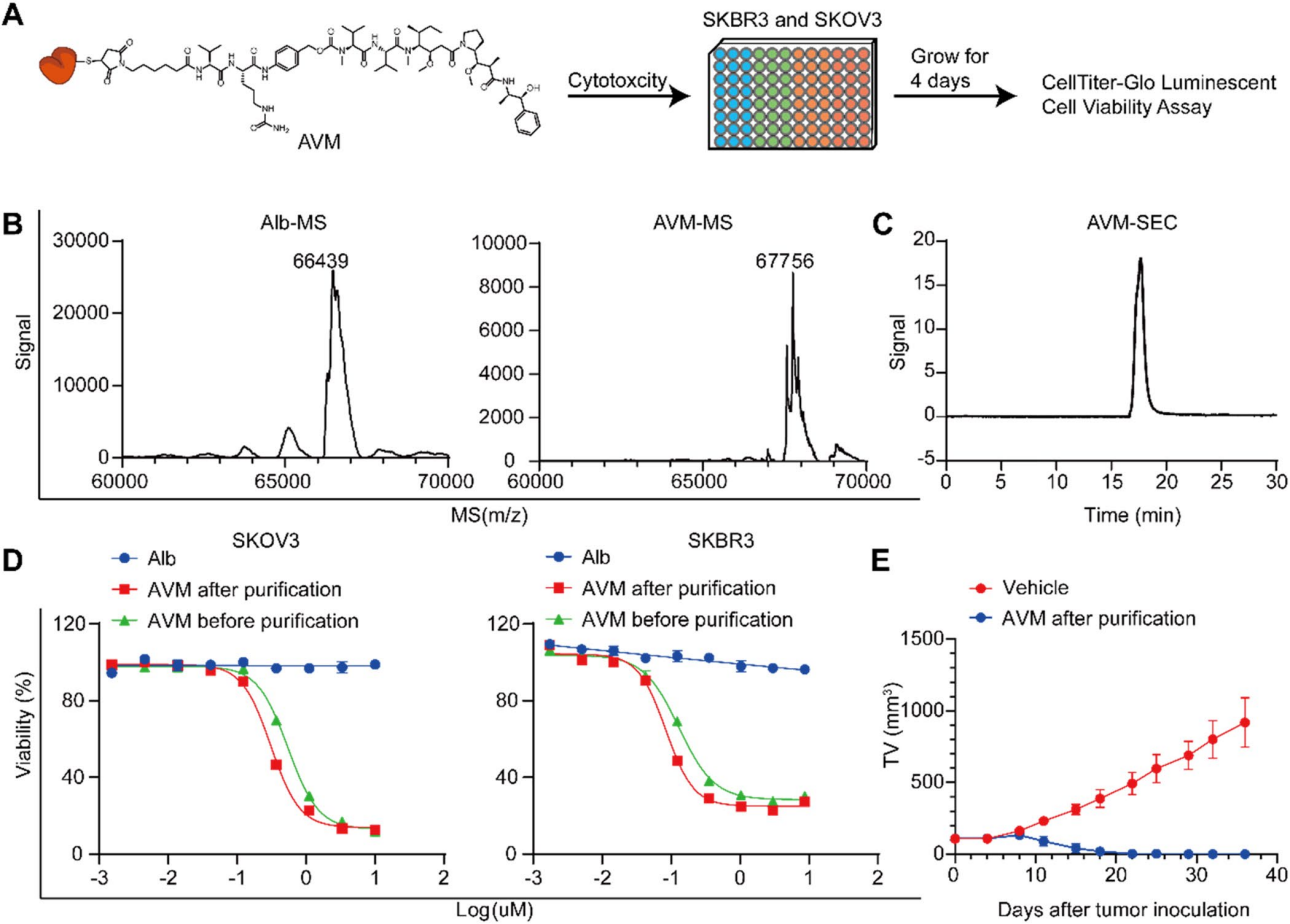
*In vitro* characterization, cytotoxicity and *in vivo* effects of conjugate AVM. A) The experimental process of cytotoxic in tumor cells; B) the mass spectrum of Alb, conjugate AVM before purification and after purification; C) the size exclusion chromatography spectrum of AVM after purification; D) Calculated EC_50_ value of Alb, conjugate AVM before purification and after purification on human ovarian cancer cells SKOV3 and human breast cancer cells SKBR3; E) *in vivo* effects of conjugate AVM after purification in xenografted mice. NOD/SCID female mice were implanted subcutaneously with SKOV3 cells. When the tumors reached ∼ 120 mm^3^, the mice were given vehicle and AVM after purification on days 0, 7, 14 and 21 respectively. Results shown as mean ± SD, *n* = 6/group.

As described above, AVM is a key intermediate. Therefore, the first study focused on the effect of different conjugate AVM qualities. Conjugate AVM has been reported in previous research (Liu et al., 2020), but a large amount of unconjugated Alb remained in the product according to the research method (approximately 50%). The instability of albumin cysteine 34 (Cys34) might be the main reason for the remaining unconjugated Alb. Accordingly, the conjugate AVM was first purified by hydrophobic interaction chromatography (Figure S2). By comparing the mass spectrum results, it was determined that almost no unconjugated Alb remained in the purified AVM (purity > 95%) (Figures 2A and S3A), indicating that unconjugated Alb was basically removed. The results of size exclusion chromatography confirmed (Figures 2B and S3B) that the purified conjugate AVM was almost composed of a single monomeric species (purity > 95%). Then, the cytotoxicity of the conjugates on SKBR3 and SKOV3 cells was determined using long-term (96 h) drug exposure assays. The EC_50_ values of conjugate AVM before and after purification ranged from 130.4 ± 14.10 nM to 83.3 ± 9.30 nM (increased by 0.64 times) and 545.6 ± 43.60 nM to 308.7 ± 23.20 nM (increased by 0.57 times), respectively (Figure 2C). Therefore, the increase in purity significantly improved cell cytotoxicity. Furthermore, in *in vivo* therapy experiments, it was determined that purified conjugate AVM can more effectively inhibit tumor growth at the same dose (47 mg/kg) given in the research (Figures 2D and S3C) (Liu et al., 2020).

**Figure 3.**
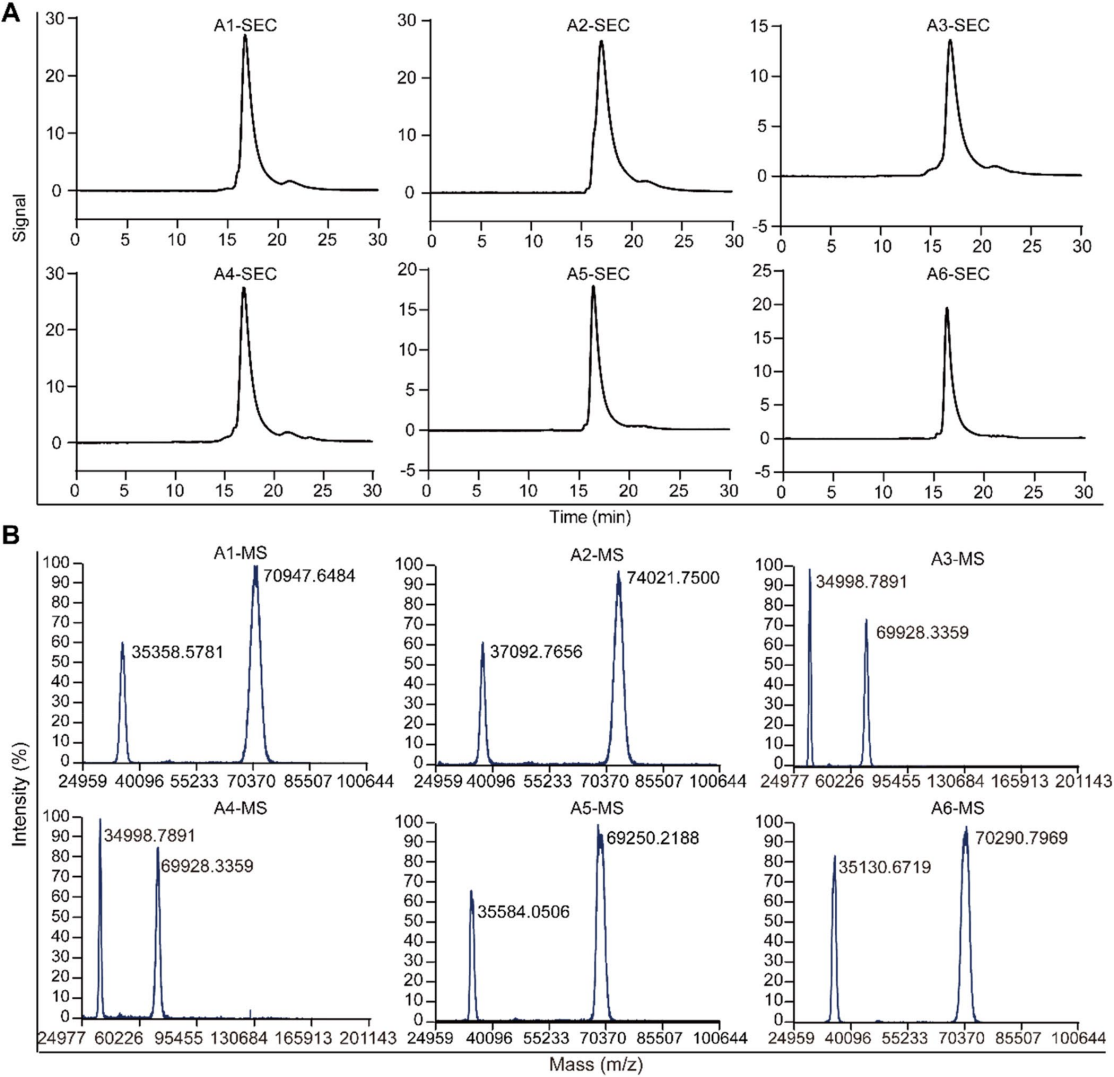
*In vitro* characterization of conjugates A1–A6. A) The size exclusion chromatography spectrum of conjugates A1–A6; B) the mass spectrum of conjugates A1–A6.

Then, on the basis of conjugate AVM with controllable quality, conjugates A1/A2 (GA modified) and conjugates A5/A6 (BA modified) were designed and synthesized. According to size exclusion chromatography analysis, all the conjugate preparations produced a nearly exclusive (> 95% purity) single monomeric species (Figure 3A). The mass spectrum results showed that the quantities of GA and BA groups linked to the modifier (Figure 3B) were determined. The quantity of conjugate A1-linked GA groups was 19, that of A2 was 38, the quantity of conjugate A5-linked BA groups was 13, and that of A6 was 28.

**Figure 4.**
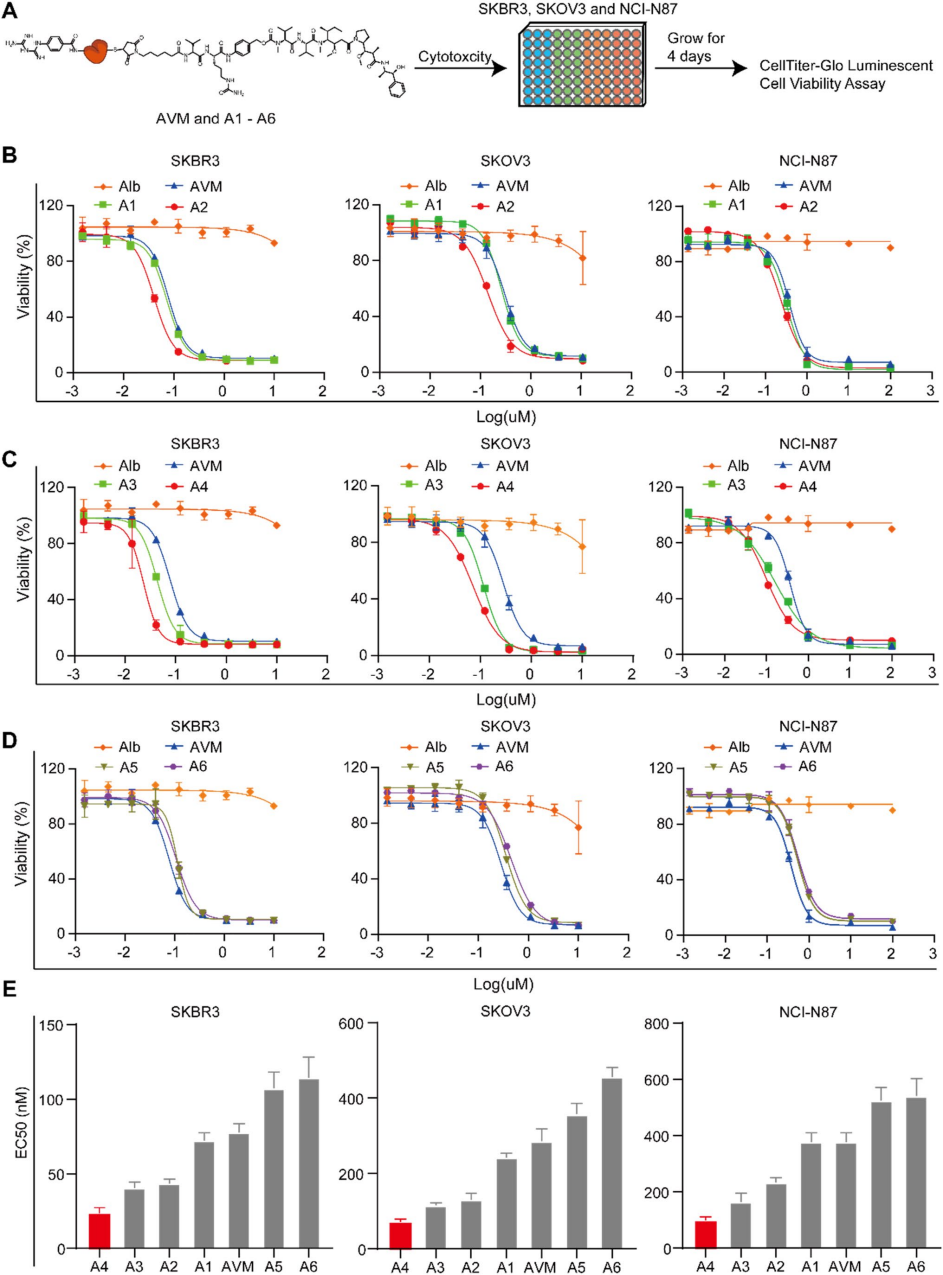
Cytotoxicity of the conjugates AVM and A1–A6 in tumor cells. A) The experimental process of cytotoxic in tumor cells; B) Calculated EC_50_ value of conjugates AVM, A1 and A2 on human ovarian cancer cells SKOV3, human breast cancer cells SKBR3 and gastric carcinoma cells NCI-N87; C) Calculated EC_50_ value of conjugates AVM, A3 and A4 on human ovarian cancer cells SKOV3, human breast cancer cells SKBR3 and gastric carcinoma cells NCI-N87; D) Calculated EC_50_ value of conjugates AVM, A5 and A6 on human ovarian cancer cells SKOV3, human breast cancer cells SKBR3 and gastric carcinoma cells NCI-N87; E) the EC_50_ value of conjugates AVM and A1-A6 on human ovarian cancer cells SKOV3, human breast cancer cells SKBR3 and gastric carcinoma cells NCI-N87.

The cytotoxicity of the conjugates on SKBR3, SKOV3 and NCI-N87 cells was determined using long-term (96 h) drug exposure assays (Figure 4B–E). The results showed that the EC_50_ values of conjugate AVM in the three cell lines were 77.61 ± 6.07 nM, 286.00 ± 33.10 nM and 376.10 ± 33.70 nM (Table 2). The EC_50_ values of conjugate A1 modified with 19 GA groups in the three cell lines were 72.25 ± 5.19 nM, 242.00 ± 12.50 nM and 322.60 ± 49.10 nM (Table 2). The cytotoxicity was not significantly different between conjugates AVM and A1. After further increasing the quantities of GA-modified (38), conjugate A2 was obtained, and its EC_50_ values were 43.55 ± 3.02 nM, 130.90 ± 16.40 nM and 233.50 ± 17.40 nM (Table 2) in the three cell lines. Compared with conjugate AVM, the cytotoxicity of A2 was increased 1.92, 2.18 and 1.61 times, indicating that increasing the quantity of modified GA could increase cytotoxicity. As a comparative study, the BA-modified conjugates A5 and A6 were used as negative controls because they could not provide additional hydrogen bonding with the cell membrane. Their cytotoxicity of the three tumor cells decreased by 107.2 0 ∼ 524.80 nM (Table 2), regardless of the change in the quantities of modifications. In conclusion, GA modification and an increase in its quantity could effectively increase the cytotoxicity of conjugate AVM against tumor cells.

**Table 2.** The EC_50_ of conjugates AVM and A1–A6.

	EC_50_ (nM)					
	SKBR3	Ratio	SKOV3	Ratio	NCI-N87	Ratio
AVM	77.61 ± 6.07	1.0	286.00 ± 33.10	1.0	376.10 ± 33.70	1.0
A1	72.25 ± 5.19	1.07↑	242.00 ± 12.50	1.09↑	322.60 ± 49.10	1.17↑
A2	43.55 ± 3.02	1.92↑	130.90 ± 16.40	2.18↑	233.50 ± 17.40	1.61↑
A3	40.38 ± 3.96	2.77↑	115.60 ± 7.30	2.29↑	163.90 ± 32.60	2.29↑
A4	23.39 ± 3.97	3.32↑	71.78 ± 7.40	3.97↑	98.09 ± 12.95	3.83↑
A5	107.20 ± 10.91	0.72↓	357.10 ± 29.10	0.80↓	524.80 ± 46.30	0.72↓
A6	114.50 ± 13.80	0.68↓	456.60 ± 25.30	0.63↓	541.20 ± 60.80	0.69↓

Inspired by the study of GA-modified albumin drug conjugates. Furthermore, BGA was introduced to form more hydrogen bonds with cell membranes, which could more effectively enhance the endocytosis ability of conjugate AVM. Therefore, conjugates A3 and A4 were obtained by modification with BGA. According to size exclusion chromatography analysis, conjugate A3 and A4 preparations produced a nearly exclusive (> 95% purity) single monomeric species (Figure 3A). The mass spectrum results showed that the number of conjugate A3-linked BGA groups was 10, and the number of conjugate A4 groups was 15 (Figure 3B). Additionally, the cytotoxicity was tested. The EC_50_ values of conjugate A3 in three cell lines, SKBR3, SKOV3 and NCI-N87, were 34.2 nM, 11.6 nM and 163.9 nM, which increased 2.27, 2.47 and 2.29 times compared with conjugate AVM, respectively (Figure 4C). Then, conjugate A4 was obtained by increasing the modified amount of BGA. The EC_50_ values of conjugate A4 in the three cell lines were 23.4 nM, 7.2 nM and 98.1 nM, which increased 3.32, 3.97 and 3.83 times that of conjugate AVM, respectively. Interestingly, the activity was significantly improved.

As described above, the activity of BGA modification was better than that of GA modification, and the cytotoxicity could also be improved by increasing the quantities of modifications.

### 3.4. Further spatial structural characterization and stability studies of conjugates A3 and A4

**Figure 5.**
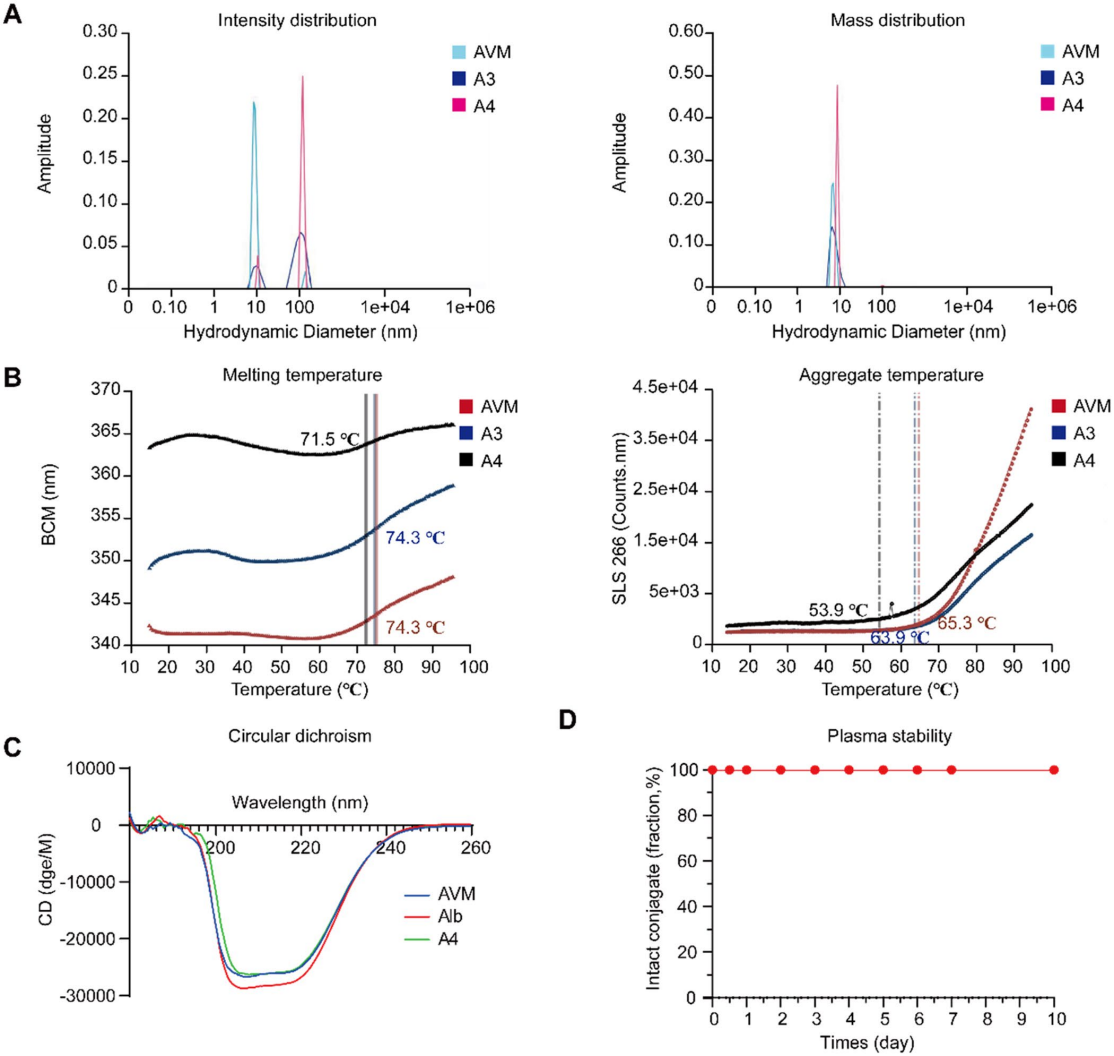
Further spatial structural characterization and stability studies of conjugates. A) The light intensity distribution and mass distribution of conjugates AVM, A3 and A4; B) the T_m_ and T_agg_ (266 nm) spectrum of conjugate AVM, A3 and A4; C) the circular dichroism of Alb, AVM and A4. D) The plasm stability of conjugate A4.

Afterwards, further spatial structural characterization and stability of conjugates A3 and A4 with the best cytotoxicity were studied. First, the particle size was measured by dynamic light scattering (Figure 5A and Table S1). Although there was aggregation in the light intensity distribution, the mass distribution was relatively uniform (Pk 1 mass ≈ 100%). The diameters of conjugates AVM, A3 and A4 were 7.27 nm, 8.54 nm and 9.20 nm, respectively (Pk 1 mode Dia. (nm)), the modification of AVM did not change its particle size significantly, and its distribution was uniform. Then, the stability of albumin drug conjugates was investigated, and the results of T_m_ (melting temperature) and T_agg_ (aggregation temperature) are shown in Figure 5(B). The T_m_ values of conjugate AVM, A3 and A4 were 74.6 °C, 74.3 °C and 71.5 °C, respectively. With increasing quantities of modification, the T_m_ value decreased slightly, but the effect was not significant. The T_agg_ values of conjugates AVM, A3 and A4 were 65.3 °C, 63.9 °C and 53.9 °C, respectively. Similarly, as the modification increased, the T_agg_ values of the three conjugates were higher than room temperature, indicating that they have a certain stability. To explore the influence on the spatial structural characterization of albumin after modification, conjugates Alb, AVM and A4 were determined by circular dichroism (Figure 5C). The results showed that although conjugates AVM and A4 were conjugated to hydrophobic small molecules, the spatial structure did not change significantly.

Furthermore, conjugate A4 is a typical albumin drug conjugate, and the basic requirement for the design was that the conjugate could remain stable. In the stability test, conjugate A4 was tested in phosphate-buffered saline (PBS) solution and 50% plasma and incubated at 37 °C for 10 days. Aliquots were taken at predetermined time points (0, 12 h, 1, 2, 3, 4, 5, 6, 7 and 10 days), and the release of free MMAE was analyzed with LC–MS/MS. After 10 days, 0.23% and 0.01% MMAE of conjugate A4 was released in PBS and 50% plasma, respectively, indicating that conjugate A4 could remain stable in PBS and 50% plasma at 37 °C (Figures 5D and S4).

### 3.5. Study on the drug release performance of conjugate A4 based on an integrated diagnosis and treatment strategy

**Figure 6.**
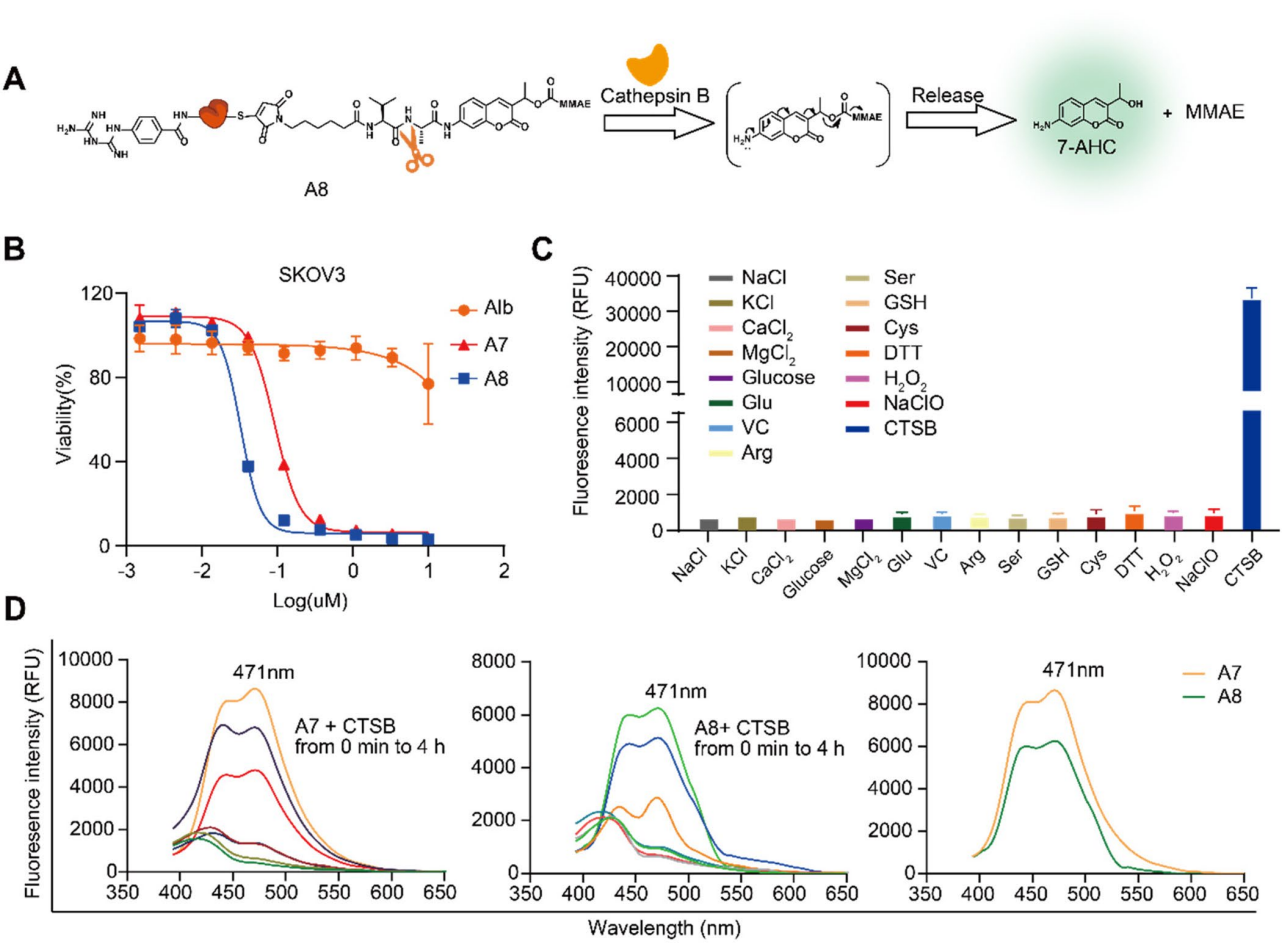
Fluorescence properties and cytotoxicity of A8. A) Cleave mechanism of conjugate A8 and fluorescence release mechanism of 7-AHC; B) Calculated EC_50_ of conjugates A7 and A8 on human ovarian cancer cells SKOV3; C) changes in fluorescence intensity of conjugate A8 at 471 nm in the presence of various species; D) the fluorescence response of 10 µM conjugate A7 with CTSB; the fluorescence response of 10 µM conjugate A8 with CTSB; the fluorescence response of 10 µM conjugates A7 and A8 with CTSB after 4 h.

Importantly, for conjugate A4, the payload can be released effectively at the target site. To study the payload release performance of conjugate A4, the PAB (log P_PAB_ = 0.66) fragment in conjugate A4 was replaced with the coumarin group 7-AHC (7-amino-3-hydroxyethyl coumarin, log P_7-AHC_ = 0.63) to design theranostic albumin drug conjugates A7 and A8. In the presence of cathepsin B (CTSB), conjugates A7 and A8 could allow self-elimination to release MMAE and activate the fluorescent Group 7-AHC (Figure 6A). Interestingly, conjugates A7 and A8 maintained cell cytotoxicity similar to conjugates AVM and A4, with EC_50_ values of 92.5 nM and 32.1 nM in SKOV3 cells, respectively. In addition, conjugate A8 has the potential to be developed into an integrated medicine for diagnosis and treatment (Figure 6B).

Then, the selectivity of conjugate A8 for CTSB over other biological species was evaluated. To assess the possibility of interference, conjugate A8 was reacted with various biological species, including NaCl, KCl, CaCl_2_, glucose, MgCl_2_, Glu, VC, Arg, Ser, GSH, Cys, DTT, H_2_O_2_, NaClO, and CTSB. As shown in Figure 6(C), increased fluorescence intensity was observed only when conjugate A8 was reacted with CTSB. No significant changes in fluorescence intensity were observed after the addition of other biological species. These findings indicated that conjugate A8 selectively reacts with CTSB. Both conjugates A7 and A8 can release strong fluorescence at 471 nm under the action of CSTB (Figure 5D), which is consistent with the characteristic emission spectrum of 7-AHC (there is maximum excitation light at 471 nm) (Zhang et al., 2019). The results also showed that the dipeptide linker was cleaved over time, and then 7-AHC and MMAE were released to play a role.

### 3.6. Study on the mechanism of action of conjugate A4

**Figure 7.**
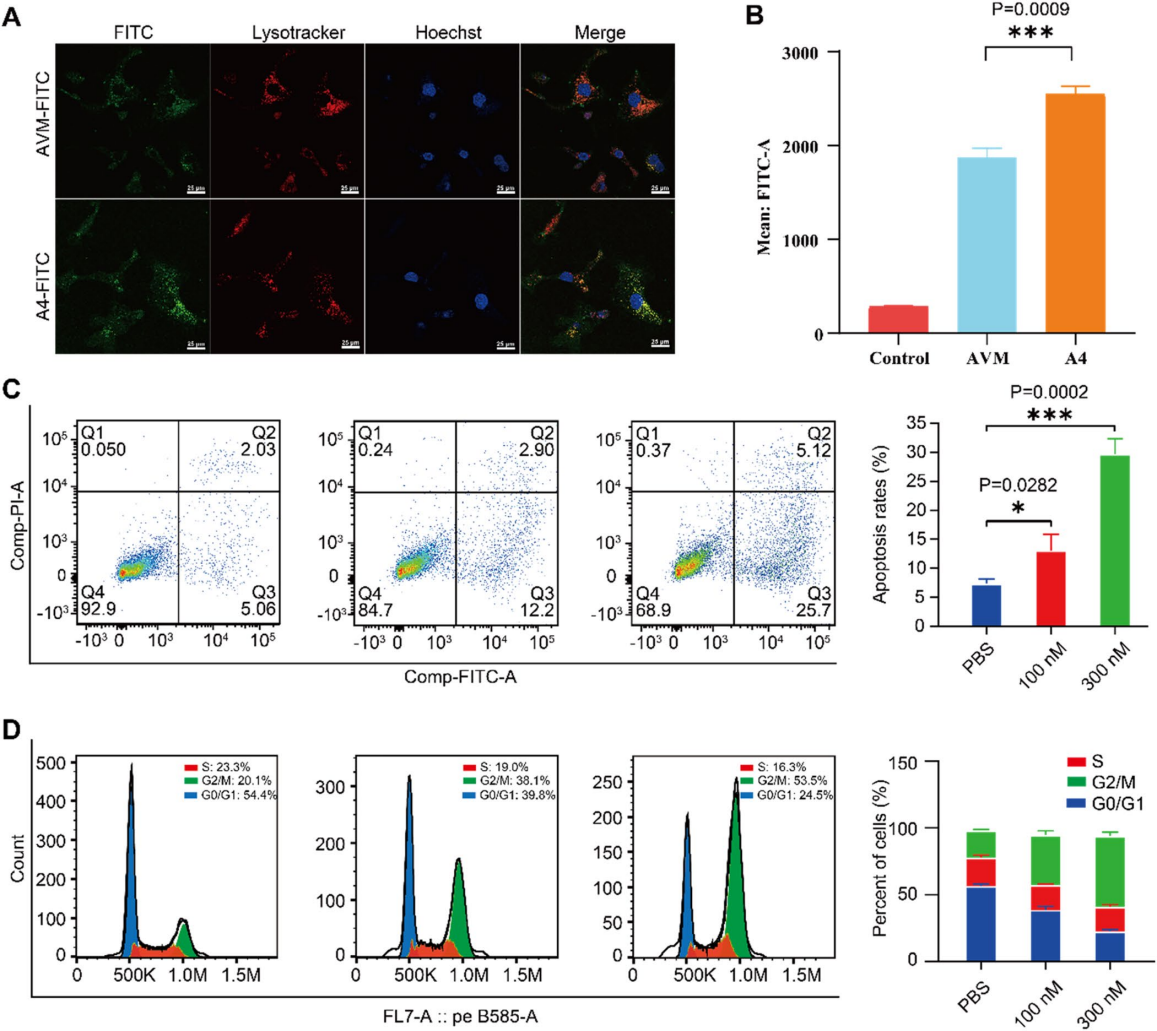
The payload internalized into lysosome was demonstrated by confocal microscope, flow cytometric analysis and apoptosis analysis. A) Confocal images of SKOV3 cells treated with conjugates AVM-FITC and A4-FITC complexes (green) for 24 h. Subcellular localization of conjugates AVM-FITC and A4-FITC in SKOV3 cells. Cells were treated with 600 nM conjugates AVM-FITC and A4-FITC for 24 h. Then LysoTracker-Red was used to identify the lysosomes and Hoechst-blue was used to identify nucleus. Scale bars: 25 µm; B) The endocytosis level of conjugates AVM-FITC and A4-FITC; C) apoptosis analysis of conjugate A4 under the concentration of 0 nM, 100 nM and 300 nM in SKOV3 cells. Data are presented as means ± SEM (*n* = 3), unpaired two-tailed *t* tests were used, and statistical analyses were performed using Prism 7.0, p*** < 0.0005, p* < 0.05. D) Cell-cycle analysis of conjugate A4 under the concentration of 0 nM, 100 nM and 300 nM in SKOV3 cells. Data are presented as means ± SEM (*n* = 3). data are presented as means ± SEM (*n* = 3), unpaired two-tailed *t* tests were used, and statistical analyses were performed using Prism 7.0, p^****^ < 0.0001, p** < 0.01.

Importantly, whether the modified conjugate AVM could be endocytosed was the key to verifying the feasibility of this new strategy. First, we used FITC to label Alb and Alb-BGA15 and characterized the endocytosis capacity of tumor cells (HeLa cells) through laser confocal experiments. The results are shown in Figure S5. The intracellular fluorescence intensity of unmodified Alb was weak, indicating that its endocytosis ability was poor. After the modification of Alb with BGA, the fluorescence intensity in the cell increased, indicating that the modified Alb could effectively enhance the endocytosis ability. Next, FITC-labelled conjugates AVM and A4 were used to characterize the endocytosis ability of tumor cells (SKOV3 cells) through laser confocal experiments. The results (Figures 7A and S5) show that conjugate AVM has weak intracellular fluorescence intensity and relatively poor endocytosis ability. After modification with BGA (conjugate A4), the endocytosis ability was significantly enhanced. Flow cytometry was used to quantify the endocytosis level of AVM-FITC and A4-FITC (Figure 7B). The results shown that, under the same conditions, the intracellular fluorescence intensity of conjugate A4-FITC was higher than that of conjugate AVM-FITC (p*** = 0.0009). In summary, the above experimental results showed that conjugate AVM modified with BGA can effectively enhance endocytosis ability.

Then, the apoptosis of conjugate A4, which has the best cell cytotoxicity, was tested. MMAE is a tubulin inhibitor that causes the cell cycle to stagnate in the G2/M phase, leading to cell apoptosis (Dumontet & Jordan, 2010). SKOV3 cells were treated with conjugate A4 at different concentrations for 48 h The results are shown in Figure 7(C). The apoptosis rates of PBS were 7.33%, but for conjugate A4 at concentrations of 100 nM and 300 nM, the apoptosis rates were 13.06% and 29.67%, respectively. Compared with the control group, the apoptosis rate was significantly increased, which indicated that conjugate A4 could release MMAE to induce cell apoptosis effectively. And the cell-cycle of conjugate A4 also tested. SKOV3 cells were treated with conjugate A4 at different concentrations for 48 h The results are shown in Figure 7(D). The G2/M percent cell of PBS and conjugate A4 at concentrations of 100 nM and 300 nM were 20.1% and 38.1% and 53.5%, respectively. The result shown that conjugate A4 could arrest the cell cycle of SKOV3 cell at G2 phase effectively.

### 3.7. In vivo potency in xenografted mice

**Figure 8.**
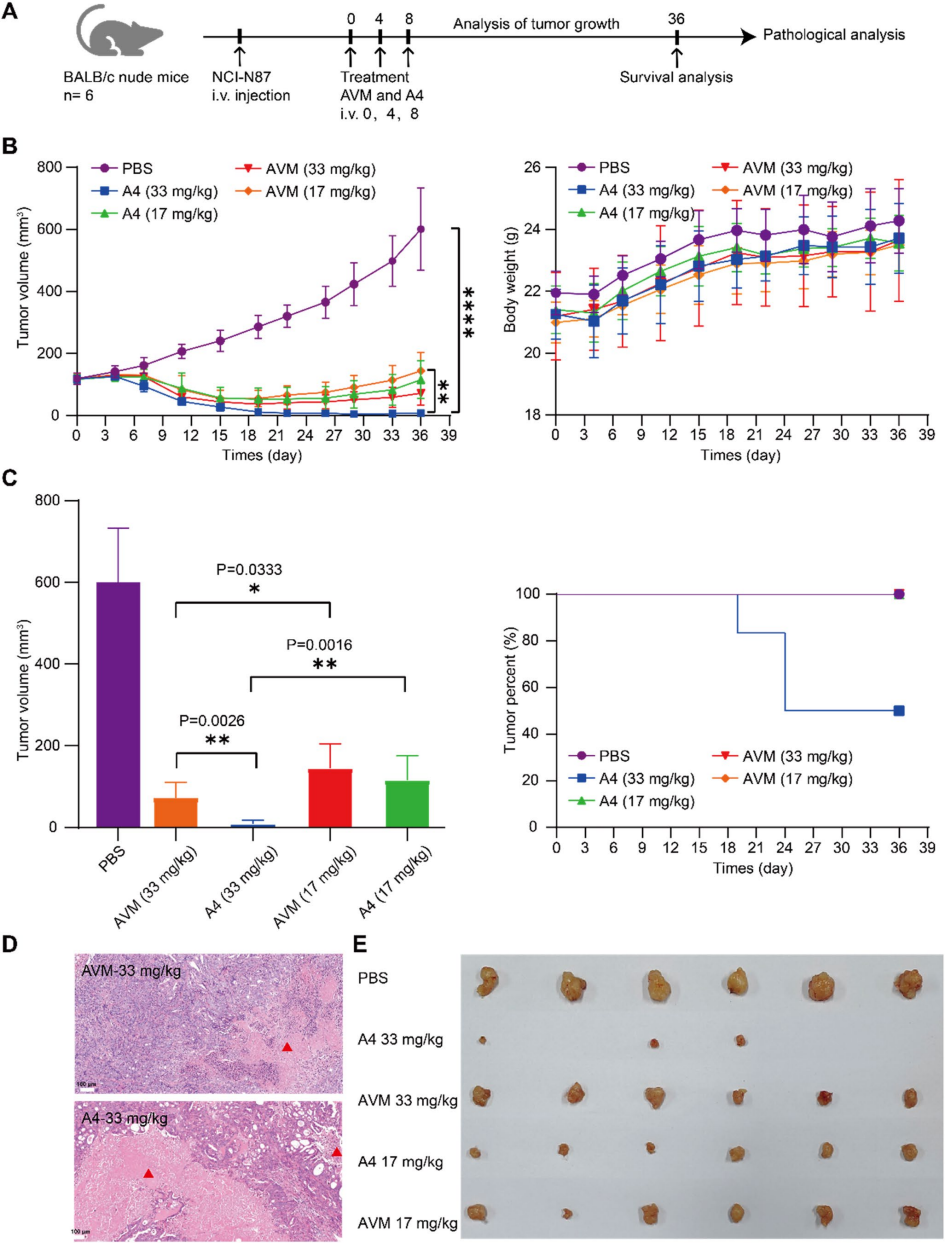
*In vivo* effects of conjugates AVM and A4 in xenografted mice. A) The experimental process of in vivo potency in xenografted mice; (B) BALB/c nude mice were implanted subcutaneously with NCI-N87 cells. When the tumors reached ∼ 180 mm^3^, the mice were given PBS, AVM, and A4 on days 0, 4 and 8 respectively. Results are shown as mean ± SD, *n* = 6/group. The tumor volume, changes in body weight and tumor weight of the test mice during the treatment and observation period. (C) To compare the difference in activity between conjugates AVM and A4, unpaired two-tailed *t* tests were used, and statistical analyses were performed using Prism 7.0. p^****^ < 0.0001, p** < 0.005, p* < 0.05. (D) Histological sections of NCI-N87 tumor tissues with H&E staining. Scale bars: 100 µm. (E) Resulting tumors excised from the animals after treatment on day 36.

Finally, the *in vivo* efficacy of albumin drug conjugates before and after modification was evaluated, and the conjugates AVM and A4 were used for tumor inhibition experiments in two groups at different doses. NCI-N87 cells were inoculated into xenogeneic BALB/c nude mice. The conjugates were administered via the tail vein on Days 0, 4 and 7 at doses of 33 mg/kg and 17 mg/kg (Figure 8A). Compared with the control group (PBS group), the four treatment groups had significant tumor inhibition. The high-dose group (33 mg/kg) treated with conjugates AVM and A4 had a significant difference in tumor volume (P** = 0.0026), and 50% of the tumors in the A4 group had completely disappeared. However, in the low-dose treatment group (17 mg/kg), there was no significant difference between the two groups (*P* > 0.05). Meanwhile, no weight loss was observed in any treatment group during the treatment period, and there was no significant difference in the weight of the test mice between the treatment groups and the control group. The results indicated that conjugates AVM and A4 were preliminarily well tolerated at the therapeutic dose administered (Figure 8B). In addition, the tumor volumes of the conjugate A4 and AVM treatment groups with different doses were significantly different (A4: p** = 0.0016, AVM: p* = 0.0333), indicating that the inhibition of tumors was dose-dependent (Figure 8C).

Then, hematoxylin and eosin (H&E) staining was used to study the histological effects of albumin drug conjugates on tumors (Figures 8D and S6). Compared with the control group (PBS group), the treatment group given albumin drug conjugates exhibited obvious tumor tissue necrosis. Among them, the tumor tissue necrosis area of conjugates AVM and A4 in the high-dose treatment group was larger than that in the low-dose treatment group. In addition, the tumor necrosis area of the conjugate A4 treatment group was larger than that of the conjugate AVM group at the same dose.

The above results indicate that there were significant differences in tumor inhibition *in vivo* between the albumin drug conjugates before and after modification. It is also important to note that the modification of albumin drug conjugates could enhance their pharmacological activities.

## 4. Discussion

In this study, to overcome the systemic toxicity and side effects of small molecule chemotherapy drugs, albumin was selected as the drug delivery system to synthesize conjugate AVM. First, conjugate AVM was purified by hydrophobic interaction chromatography, and its quality and *in vitro*/*vivo* potency were significantly improved. However, the *in vitro*/*vivo* potency of conjugate AVM was affected by its poor endocytosis ability. Guanidine modification of conjugate AVM could enhance endocytosis ability by forming hydrogen bond interactions with the cell membrane. Therefore, modified conjugates A1–A6 were synthesized. Among them, conjugate A4, with the best endocytosis ability, could increase the *in vitro* potency by approximately 4 times compared with the unmodified conjugate AVM and could effectively inhibit the growth of tumors at 33 mg/kg. Then, the particle size, T_m_ and T_agg_ were measured by dynamic light scattering (DLS) to further control the quality of conjugates A3 and A4. The results showed that conjugates A3 and A4 had a certain stability. Additionally, the circular dichroism test showed that the spatial structure of the modified conjugate A4 did not change significantly compared with Alb and the conjugate AVM.

To further determine that albumin drug conjugates could effectively release payload in tumor cells, theranostic conjugates A7 and A8, which were integrated with diagnosis and treatment, were synthesized. Finally, the effectiveness of payload release was verified by fluorescence and cytotoxicity experiments.

The endocytosis ability of conjugates by the cell membrane is also important, which determines the effectiveness of macromolecular drug conjugates. The laser confocal experiment showed that the modified conjugate A4 could effectively increase endocytosis ability compared with the unmodified conjugate AVM. Importantly, the colocalization of Lysotracker and FITC showed that the modified conjugate A4 could effectively enter the lysosome. The apoptosis and cell-cycle experiment showed that the modified conjugate A4 could effectively release MMAE to arrest the cell cycle at G2 phase and induce apoptosis of tumor cells.

Finally, the structure-activity relationship was summarized: 1. For modified albumin drug conjugates, the modified group BGA was better than GA which reflected in improving the endocytosis ability and *in vitro*/*vivo* potency; 2. The increased quantities of albumin modifiers would also enhance the endocytosis ability; 3. The BA group cannot enhance endocytosis ability because it could not provide additional hydrogen bond action. Finally, it was concluded that the guanidine-modified albumin drug conjugates could increase the *in vitro*/*vivo* potency through the additional hydrogen bond to enhance endocytosis ability.

This work might provide new ideas for optimizing the design of drug carriers by enhancing their endocytosis ability and biological activity. However, the modified group quantities of the conjugates failed to effectively remain the same. It is also regrettable that conjugates with more modified groups were not synthesized to summarize the structure-activity relationship more accurately. In the future, chemical synthesis could be used to optimize conjugate conditions, and different structures and quantities of modified groups could also be explored on the bioavailability of albumin drug conjugates.

## 5. Conclusions

To address the problem of insufficient endocytosis ability of traditional albumin drug conjugates, this paper reports a new strategy to enhance endocytosis ability for the first time. Through the study of modified albumin drug conjugates with different structures and quantities, the preferred conjugate A4 was obtained. After testing, the *in vitro/vivo* potency of conjugate A4 was more effective than that of the unmodified conjugate AVM. In addition, this work was the first to design the theranostic albumin drug conjugate A8, which can quickly and intuitively realize the study of the drug release performance of the conjugate. The strategy of guanidine-modified albumin drug conjugates proposed in this work could provide new ideas for the development of new generational albumin drug conjugates.

## Supplementary Material

Supplemental Material

## Data Availability

The authors confirm that the data supporting the findings of this study are available within the article [and/or] its supplementary materials.
