# Supplementary material for: Guanidine-modified albumin-MMAE conjugates with enhanced endocytosis ability
Source: Drug Deliv. 2023 Jul 11;30(1):2219433. doi: 10.1080/10717544.2023.2219433 (PMC10339779; doi:10.1080/10717544.2023.2219433)
Supplement: Supplemental Material [file IDRD_A_2219433_SM0184.docx]

**Support information**

**Scheme 1.** Synthesis of E-15C. Reagents and conditions: (a) ethyl acetoacetate, piperidine, EtOH, reflux, 1.5 h; (b) NaBH_4_, CeCl_3_, MeOH/THF = 1:1, 0 ºC, 1.5 h; (c) FeCl_3_, N_2_H_4_·H_2_O, C, EtOH, reflux, 2 h; (d) amino coumarin, T3P, NMM, THF, 0 ºC, 3.5 h; (e) DMF, piperidine, RT, 1 h; (f) 6-maleimidohexanoic acid N-hydroxysuccinimide ester, DIPEA, DMF, RT, 12 h; (g) Bis(4-nitrophenyl) carbonate, DIPEA, DMF, RT, 12 h; (h) MMAE, DIPEA, HOBT, DMF, 12 h;


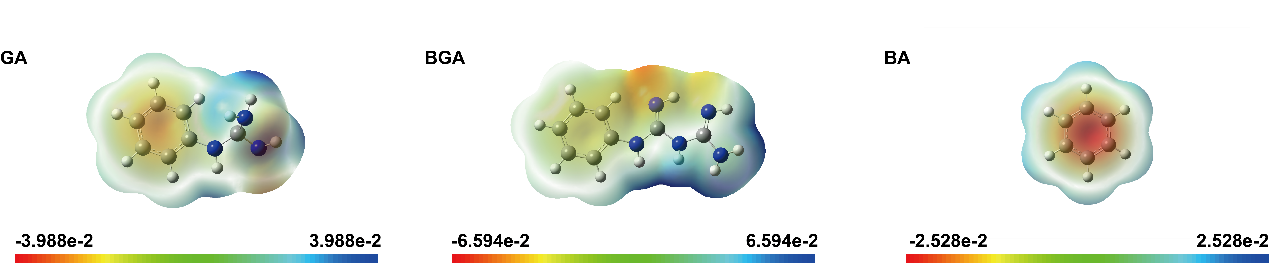


**Figure S1**. The electrostatic potential maps of BGA, GA and BA.

**The molecular electrostatic potential surfaces**

GaussView was used to construct the molecular structure of benzene, and then the structures were optimized at DFT level with B3LYP function and 6-311G (d,p) basis set implemented in Gaussian 09 software program for obtain the most stable conformation. The above structure is used as the initial structure for single point energy calculation under the basis set as well. Subsequently, electrostatic potential surfaces were performed at the same method.


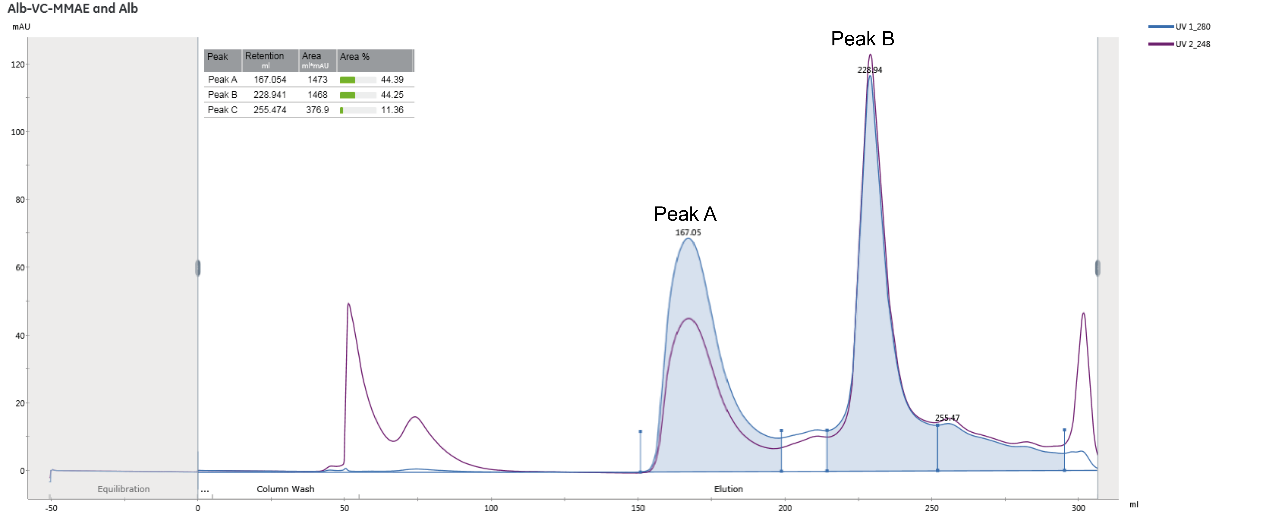


**Figure S2**. The HIC spectrum of separated Alb (Peak A) and Alb-VC-MMAE (Peak B).


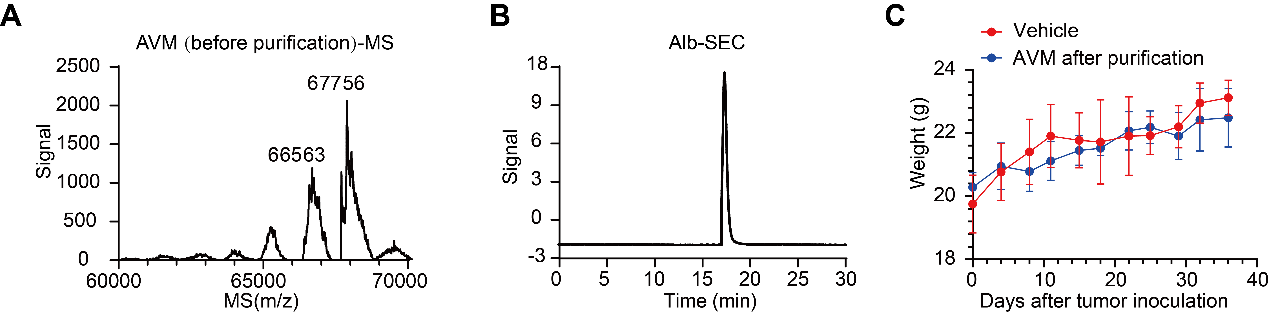


**Figure S3**. A) The mass spectrum of AVM before purification; B) The size exclusion chromatography spectrum of Alb; C) The changes in body weight of the mice during the treatment and observation period.

**Table S1**. The dynamic light scattering (DLS) data of AVM, A3, A4 and IgG.

| Sample | Z-Ave.Dia.  (nM) | PDI | Fit Var. | Pk 1 mode Dia. (nm) | Pk 1 polydispersity (%) | Pk 1 mass (%) | Pk 2 mode Dia. (nm) | Pk 2 polydispersity (%) | Pk 2 mass (%) |
| --- | --- | --- | --- | --- | --- | --- | --- | --- | --- |
| AVM | 7.59 | 0.117 | 5.844E-05 | 7.27 | 18.70 | 100 | 143.23 | 16.32 | - |
| A3 | 43.37 | 0.399 | 0.019 | 8.54 | 24.66 | 99.45 | 110.42 | 31.40 | 0.55 |
| A4 | 93.4 | 0.396 | 1.948E-03 | 9.20 | 21.71 | 98.34 | 122.40 | 23.00 | 1.66 |
| IgG | 12.51 | 0.112 | 2.428E-05 | 14.72 | 34.38 | 100 | - | - | - |


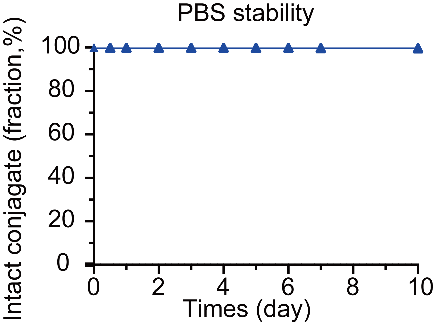


**Figure S4**. The PBS stability of conjugate A4.


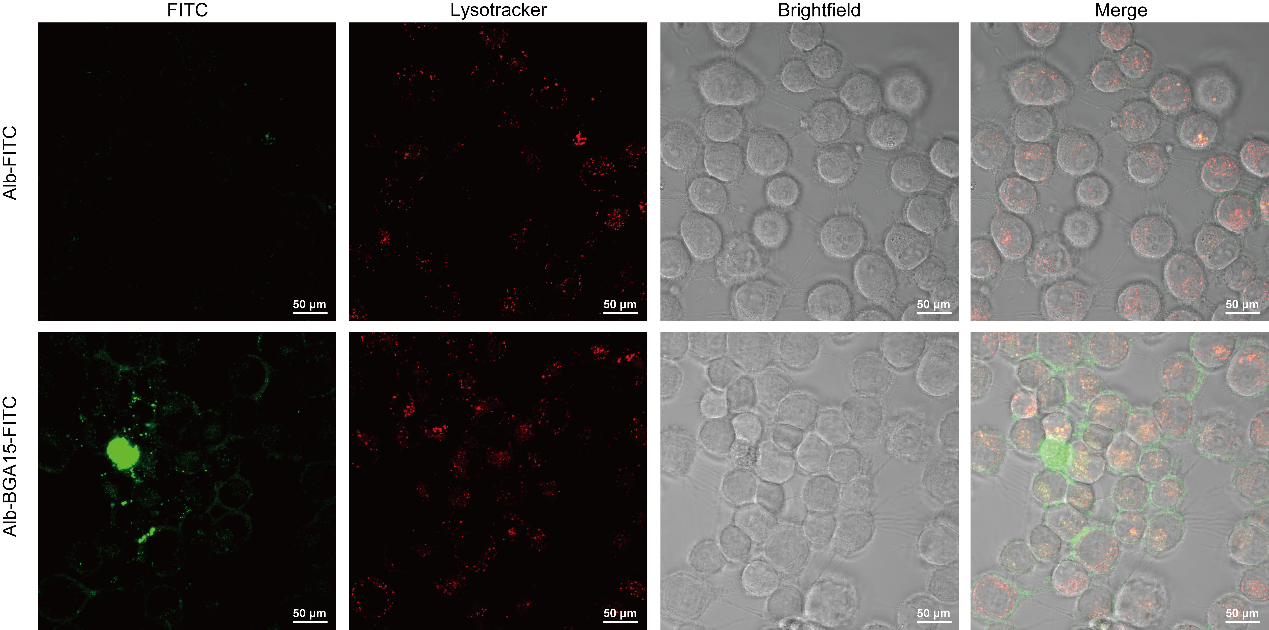


**Figure S5**. Confocal images of HeLa cells treated with Alb-FITC and Alb-BGA15-FITC complexes (green) for 24 h. Subcellular localization of Alb-FITC and Alb-BGA15-FITC in SKOV3 cells. Cells were treated with 600 nM Alb-FITC and Alb-BGA15-FITC for 24 h. Then LysoTracker-Red was used to identify the lysosomes. Scale bars: 50 µm;


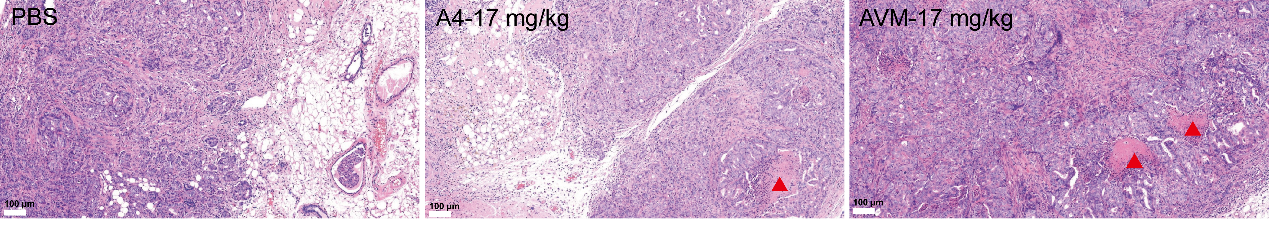


**Figure S6**. Histological sections of NCI-N87 tumor tissues with H&E staining. Scale bars: 100 µm.
